# DNA-barcoding and a new data about the karyotype of *Myotis petax* (Chiroptera, Vespertilionidae) in the Russian Far East

**DOI:** 10.3897/CompCytogen.v14i4.54955

**Published:** 2020-10-19

**Authors:** Uliana V. Gorobeyko, Irina V. Kartavtseva, Irina N. Sheremetyeva, Denis V. Kazakov, Valentin Yu. Guskov

**Affiliations:** 1 Federal Scientific Center of the East Asia Terrestrial Biodiversity Far Eastern Branch of Russian Academy of Sciences, Vladivostok, Russia East Asia Terrestrial Biodiversity, Far Eastern Branch of Russian Academy of Sciences Vladivostok Russia; 2 Institute of Environmental and Agricultural Biology (X-BIO), Tyumen State University, Tyumen, Russia Tyumen State University Tyumen Russia

**Keywords:** bats, chromosome, COI, heterochromatin, *
Myotis
*, NOR

## Abstract

The DNA-barcoding and chromosomal study of the eastern water bat, *Myotis petax* Hollister, 1912, from the earlier unexplored localities in the Russian Far East are carried out. The COI barcoding obtained for 18 from a total of 19 individuals captured in five localities in the Russian Far East showed the low nucleotide variability with the prevalence of the central, the most abundant haplotype. The chromosomal characteristics of eight *M. petax* specimens (2n = 44, NFa = 52) in the Russian Far East are clarified. The number and localization of NOR in karyotype of *M. petax* is described at the first time and differ from distributional patterns of NOR in the sibling species *M. daubentonii* Kuhl, 1819 that can be used as diagnostic feature. The considerable intraspecific variability in the distribution of heterochromatin material revealed is not typical of the genus *Myotis*, but it has been found in other species of the family Vespertilionidae.

## Introduction

The eastern water bat, *Myotis petax* Hollister, 1912, is a common Eastern Palaearctic bat species. The range of *M. petax* includes the near-water habitats throughout forest, forest-steppe and steppe zones from Western Siberia to the Russian Far East (including Sakhalin and the Kuril Islands) and, outside of Russia – in northern Mongolia, NE China, Korea and Japan (Kruskop 2012). It was first described as a distinct species from the village Kosh-Agach in the Altai Mountains (Hollister 1912). However, starting from Ognev (1928) and until the early 2000s, *M. petax* had been considered as part of the widespread polytypic species *Myotis daubentonii* Kuhl, 1819 which had included about 3 to 6 subspecies according to various estimates (Kuzaykin 1950; Gromov 1963; Tiunov 1984, 1997; Yoshiyuki 1989; Bogdanowicz 1994; Koopman 1994).

The morphological heterogeneity and the presence of two major groups of forms in *M. daubentonii* complex: the “western” and the “eastern” (including the Altai form *M. d. petax*) has been shown by Kruskop (2004). The species rank of *M. petax* was finally confirmed by the genetic and morphological data with the using SINEs as genetic markers. A total of 6 specimens of *M. daubentonii* and 7 specimens of *M. petax* (including only one bat from the Far East) were examined by molecular method (Matveev et al. 2005). It was shown by the molecular studies based on cyt b and ND1 sequences that *M. petax* is closer related to *M. macrodactylus* (Temminck, 1840), *M. pilosus* Peters, 1869 and *M. fimbriatus* Peters, 1871 than *M. daubentonii*. The closest related species for *M. daubentonii* are *M. bechsteinii* Kuhl, 1817, *M. longicaudatus* Ognev, 1927 and *M. frater* G. Allen, 1823 (Kawai et al. 2002, 2003; Ruedi et al. 2013; Ruedi et al. 2015).

The DNA-barcoding based on 657 bp length sequences of cytochrome c oxidase I (COI) gene has been studied for the 23 *M. petax* individuals including 6 specimens from the Far East, i.e. 5 bats from Sakhalin and 1 animal from the Primorsky Krai. It was revealed that the intraspecific distances for *M. petax* are amounted to 0.28% to 1.16% while interspecific distance between *M. petax* and *M. daubentonii* is 12% (Kruskop et al. 2012). The differences between cyt b sequences of *M. petax* from the Far East (n = 1) and China (n = 17) were amounted to 0.2% (Wang et al. 2010). In addition, the partial sequence of control region for one specimen from China had been studied (Zhang et al. 2009) and the full mitochondrial genomes of 4 individuals from South Korea had been obtained (Hwang et al. 2016). Otherwise, the genetics of *Myotis petax* in Far Eastern populations still remains poorly studied.

Karyotype features are essential diagnostic characteristics of many mammalian species (Vorontsov 1958; Matthey 1973; Orlov and Bulatova 1983; Mazzoleni et al. 2018). The chromosomal data are successfully applied to clarify species affinity and interspecific relationships between species of the order Chiroptera (Volleth 1987; Volleth and Heller 1994, 2012; Volleth et al. 2001; Kearney et al. 2002; Volleth et al. 2006). It was shown by our review that the karyology of Far Eastern bats is studied insufficiently (Gorobeyko and Kartavtseva 2019).

For the genus *Myotis* Kaup, 1829 the position and number of the nucleolus organizer regions (NORs) and the amount and location of heterochromatic material on chromosomes are species-specific characteristics (Harada and Yosida 1978; Ando et al. 1980; Volleth 1987; Ando et al. 1987; Ono and Obara 1994; Volleth and Heller 2012). The NOR distribution has been studied for 4 out of 6 Far Eastern *Myotis* species and varied from 5 to 13 centromeric NORs on the acrocentric chromosomes (Ono and Obara 1994). Only 3 NORs were found in karyotype of *M. daubentonii* on acrocentric pairs Nos. 8 to 10 (Volleth 1987; Volleth and Heller 2012). It is likely that the number and location of NOR on the *M. petax* and *M. daubentonii* chromosomes should be different. A small intercalary heterochromatic band in the proximal part of the long arm of X chromosome and largely heterochromatic submetacentric Y chromosome was detected in *Myotis daubentonii* karyotype (Volleth and Heller 2012). The pattern of heterochromatic material on *M. petax* chromosomes is still unknown.

Only conventional staining karyotypes of *M. petax* have been studied from the Primorsky Kray, Russian Far East (Korablev et al. 1989), and from South Korea (Yoo and Yoon 1992). The diploid number of *M. petax* did not differ from other *Myotis* species (2n = 44), but the number of autosomal arms (NFa) was different in two works and amounted to 50 or 52, respectively. The feature of genus *Myotis* the fundamental number of autosomal arms is 52 due to the short euchromatic arms on the autosomal pair No. 7 (Volleth and Heller 1994, 2012)

Thus, the aim of present paper is to study DNA barcodes and chromosomes of *Myotis petax* from the localities of the Russian Far East that are not covered by previous studies, and to compare obtained results with these data for the species. It is important to investigate the position and number of the NORs and the amount and location of heterochromatic material on chromosomes to clarify chromosomal characteristics of *M. petax* and to find the differences with the karyotypes of other *Myotis* species.

## Materials and methods

There are 19 specimens of *M. petax* caught in the Primorsky Krai (n = 7), Khabarovsky Krai (n = 4), Amur Oblast (n = 8) studied in this paper. Bats were caught using mist nets (6–7 m × 2.5 m, Ecotone, Poland) in swarming site and near summer roosts, handling in hibernation sites. The geographical origin of the examined animals and coordinates listed in Table 1. The other collecting data see Suppl. file 1. The samples used in the present study are deposited in the Genetic Mammalian Tissue Collection of the Federal Scientific Center of the East Asia Terrestrial Biodiversity, Far East Branch, Russian Academy of Sciences (Vladivostok, Russia). All applicable international, national and institutional ethics statements when using animals in research have been followed.

In addition, the 26 COI sequences of *Myotis petax* (Table 1) and 19 COI sequences of five Far Eastern *Myotis* species (*M. macrodactylus*, *M. longicaudatus*, *M. bombinus* Thomas, 1906, *M. ikonnikovi* Ognev, 1912, *M. sibirica* Kastschenko, 1905) and *M. daubentonii* from GenBank were analyzed. The COI sequence of *Murina hilgendorfi* Peters, 1880 was used as outgroup in phylogenetic analysis.

*M. macrodactylus*: HQ580337, HQ580338 (International Barcode of Life, 2010), KT862813, KT862814 (GenBank), *M. longicaudatus*: JF442982, JF442983, JF442989 (Kruskop et al. 2012), *M. bombinus*: HQ580336 (International Barcode of Life, 2010), JF442874, JF442876 (Kruskop et al. 2012), *M. ikonnikovi*: HQ974651, HQ974652 (International Barcode of Life, 2010), JF442993 (Kruskop et al. 2012), *M. sibirica*: JF442902, JF442905, JF442926 (Kruskop et al. 2012), *M. daubentonii*: JF442939, JF442942, JF442943 (Kruskop et al. 2012), and *Murina hilgendorfi*: JF442830 (Kruskop et al. 2012).

**Table 1.** Sampling localities and GenBank sequencing data of *Myotis petax*. specimen – the number of animal in Genetic Mammalian Tissue Collection of the FSCEATB FEB RAS or in Collection of Zoological Museum of Moscow University. 2n/ NFa – the diploid number of chromosome and the fundamental number of autosomal arms, X and Y – morphology of sex chromosomes: M – metacentric, SM – submetacentric, M-SM – biarmed chromosome, A – acrocentric, conv – conventional staining.

Code	Locality	Coordinates	GenBank	Specimen	Sex	2n/ NFa	X	Y	Chromosomal stainings
**1**	Primorsky Krai, Primorsky Velican Cave	43°17.133'N, 133°36.8'E	MT383996	3240	m	-			
			MT383997	3400	f	44/52	M-SM	-	conv, GTG, AgNOR
			MT383998	3865	f	44/52	M-SM	-	GTG, CBG
			–	3867	m	44/52	M-SM	A	GTG, CBG
			MT383999	3869	f	-			
**2**	Primorsky Krai, Spasskaya Cave	44°34.883'N, 132°46.083'E	MT384000	3258	m	44/52	M-SM	A	conv, AgNOR
			MT384001	3259	m	44/52	M-SM	A	conv, GTG, CBG, AgNOR
**3**	Khabarovsky Krai, Komsomolsk Nature Reserve	50°50.1'N, 137°28.7'E	MT384002	UG16-18	m	-			
			MT384004	UG21-18	m	-			
**4**	Khabarovsky Krai, Komsomolsk-on-Amur City	50°42.114'N, 137°12.291'E	MT384003	UG28-18	m	-			
			MT384005	UG36-18	f	-			
**5**	Amur Oblast, Zeya City	53°41.767'N, 127°4.317'E	MT384006	3332	m	-			
			MT384007	3333	m	44/52	M-SM	A	conv
			MT384008	3334	f	-			
			MT384009	3335	f	-			
			MT384010	3336	f	44/52	M-SM	-	conv, CBG, AgNOR
			MT384011	3337	m	-			
			MT384012	3338	f	44/52	M-SM	-	conv, GTG, CBG, AgNOR
			MT384013	3339	f	-			
**GenBank sequencing data of *Myotis petax***									
**Code**	**Locality**	**Coordinates**	**GenBank**	**Specimen**	**Sex**	**Reference**			
**6**	Primorsky Krai, Priiskovaya Cave	44°22.767'N, 133°12.283'E	JF443025	S173255	m	Kruskop et al. 2012			
**7**	Sakhalin Oblast	46°22.3'N, 141°52.217'E	JF443019, JF443032–JF443035	S175221-25	-	Kruskop et al. 2012			
**8**	Transbaikal Krai	53°22.5'N, 121°10.38'E	JF443026	S182081	m	Kruskop et al. 2012			
**9**	Transbaikal Krai	53°25.2'N, 120°19.8'E	JF443028	S175362	m	Kruskop et al. 2012			
**10**	Mongolia	47°5.783'N, 102°46.38'E	JX008075–JX008077	S187466-68	-	Kruskop et al. 2012			
**11**	Tuva Republic	50°02'N, 95°04'E	JF443020, JF443029–JF443031, JF443036– JF443038	S167627, S167738, S168602-03, S168637, S168648-49	-	Kruskop et al. 2012			
**12**	Altai Republic	51°22.2'N, 84°43.8'E	JF443024	S171621	m	Kruskop et al. 2012			
**13**	Altai	51°21.9'N, 84°42.9'E	JF443021	S171624	f	Kruskop et al. 2012			
**14**	Altai	51°17.22'N, 84°43.92'E	JF443039, JF443040	S184155-56	2f	Kruskop et al. 2012			
**15**	South Korea	36°31'N, 127°48'E	KT199099–KT199102	KW001-004	-	Hwang et al. 2016			

### DNA extraction, amplification and sequencing

Total DNA was isolated from ethanol-fixed tissues by the method of saline extraction (Aljanabi and Martinez 1997). For the DNA-barcoding we used the part of COI from 49 to 705 nucleotides, 657 bp length. The COI gene was amplified and sequenced by polymerase chain reaction and sequenced using the forward MPCO+ (5’-ATTTGCAATTCAATGTGTATT-3’) and reverse MPCO- (3’-ATAGCTCATACCATTCCTAT-5’). The both primers were designed in this study. Amplification was carried out in a 25 μL reaction mixture, which included 3–4 μg of total DNA, 2.5 μL 10× buffer, 2.5 μL of 20 mM dNTP mixture, 2 μL of each primer, 0.5 units Taq-polymerase (Sibenzim, Russia), and deionized water. The COI gene was amplified under the following conditions: 5 min DNA denaturation at 95 °C, 35 cycles of amplification (95 °C for 10 s, 47.5 °C for 60 s and 72 °C for 60 s) and 7 min chain completion at 72 °C. PCR products were purified and sequenced with the forward and reverse primers using the Big Dye Terminator series 3.1 kit (Applied Biosystems, United States). The nucleotide sequences were analyzed with the ABI Prizm 3130 sequencer (Applied Biosystems, United States) in the Federal Scientific Center of the East Asia Terrestrial Biodiversity, Far East Branch, Russian Academy of Sciences (Vladivostok, Russia).

### Phylogenetic analysis

All sequences were aligned using the software program BioEdit, version 7.0.9.0 and deposited in the GenBank database. The accession numbers of our and sequences obtained from GenBank are reported in the Table 1.

The interspecific nucleotide diversity (π) and haplotype diversity (*P*) were calculated using DnaSP6 (Hall 1999). A search for the best model of nucleotide evolution was performed using Modeltest: Hasegava-Kishino-Yano including invariant sites (HKY+I) (Nei and Kumar 2000, Kumar et al. 2018). The phylogenetic analysis was based on Maximum Likelihood (ML) method and run in MEGA-X 10.1.7 with 1000 bootstrap replicates (Kumar et al. 2018). To calculate pairwise genetic p-distances the MEGA-X 10.1.7 software used. To construct the haplotype network by the “median joining” method the Network 10 software used (https//www.fluxus-engineering.com).

### Chromosomal analysis

Chromosome preparations were obtained from *in vivo* bone marrow method (Ford and Hamerton 1956), as well as from short-term cell cultures established from spleen and bone marrow (Graphodatsky and Rajabli 1988). GTG-banding procedure was carried out according to Seabright (1971). Chromosomes were numbered using Bickham’s scheme, in which ordinal numbers have been given to all of the autosomal arms based on GTG-banding patterns (Bickham 1979). The locations of nucleolus organizer regions (NORs) were detected by sequential using of silver staining method (Bloom and Goodpasture 1976) and GTG-banding of chromosomes. Heterochromatic material was detected using C-banding (Sumner 1972). To determine the locations of heterochromatic bands on chromosomes, we used sequential GTG-staining and C-staining. The mean value of active NORs per chromosomal pair and cell was calculated according to Volleth (1987), where each distinct NOR was counted as 1.0 and indistinct one as 0.5. The greatest possible value of NORs per chromosomal pair and cell was 2.0 (Volleth 1987).

The results of differential staining were analyzed with an AXIOSKOP 2 Plus microscope (Zeiss). The microimage registration and adjustment was performed with a CCD camera with software (META Systems GmbH, Germany) of the Joint-Use Center «Biotechnology & Genetic Engineering» in the Federal Scientific Center of the East Asia Terrestrial Biodiversity Far East Branch Russian Academy of Sciences (Vladivostok, Russia).

## Results and discussion

### DNA-barcoding and phylogenetic analysis

**Figure 1.**
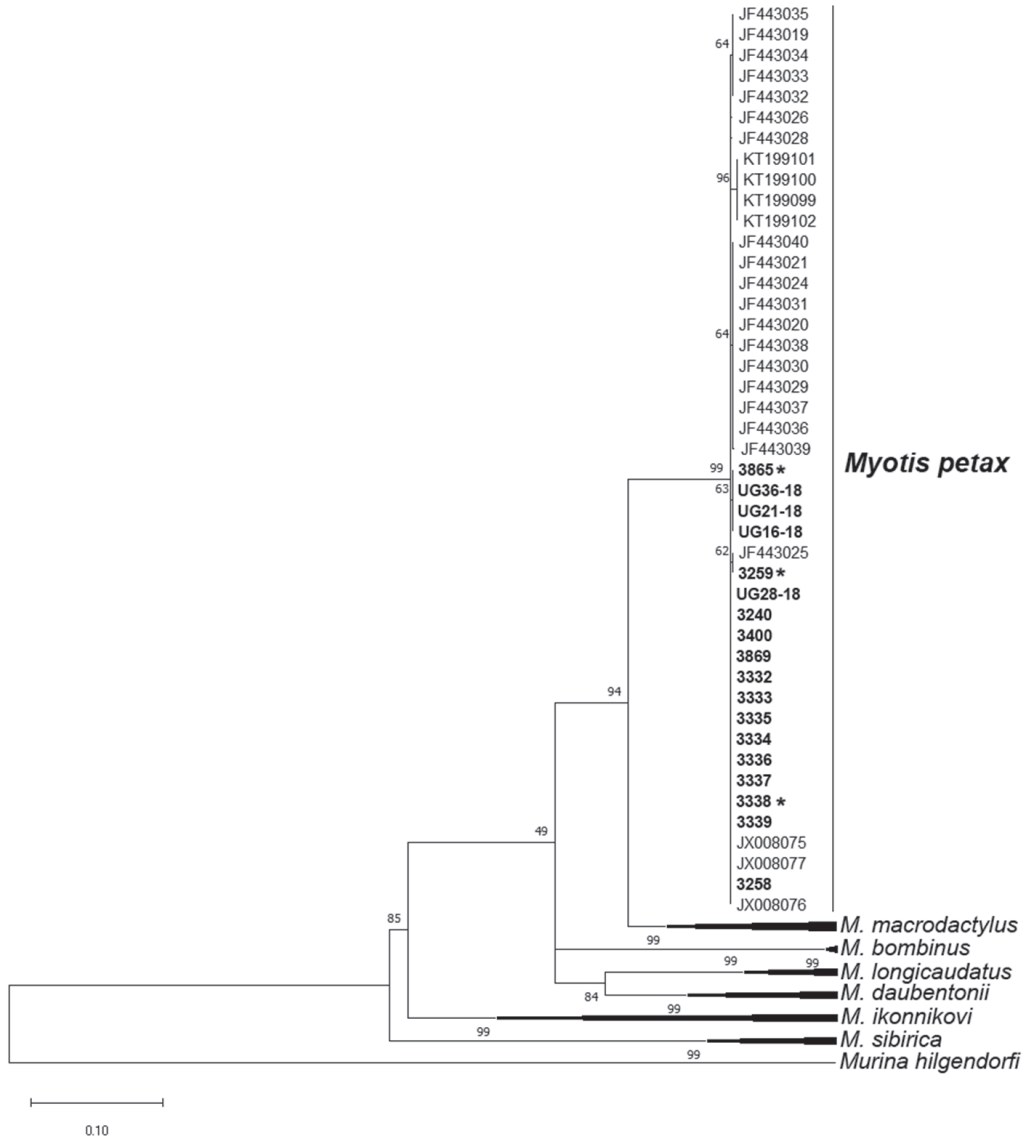
Maximal Likelihood tree of the cytochrome oxidase I gene. ML tree based on 64 COI sequences of *Myotis* species and outgroup. The bold numbers marked our data. Asterisks marked individuals for which the CBG-banding karyotype is studied.

The DNA barcodes are obtained for 18 from a total of 19 *M. petax* individuals captured in five localities in the Russian Far East. To identify the species the sequences have compared with the 45 DNA barcodes of 7 *Myotis* species (*M. petax*, *M. macrodactylus*, *M. longicaudatus*, *M. bombinus*, *M. ikonnikovi*, *M. sibirica*, *M. daubentonii*) from GenBank. All of the obtained sequences have highest similarity with sequences of *M. petax* from GenBank (Fig. 1).

A pairwise genetic distances between the specimens of *M. petax* studied vary from 0 to 0.8%. The obtained values are within the range of interspecific distances (0.28–1.16%) previously described for *M. petax* (Kruskop et al. 2012). A mean genetic p-distances between the individuals from the Primorsky Krai and South Korea is 0.54% (less than 1000 km), while a mean genetic p-distances between the Altai Mountains and the Primorsky Krai specimens is only 0.26% (approximately 3000–3500 km). This means that a geographically closer South Korean population is genetically more distant from the population of *M. petax* in the Primorsky Krai.

The nucleotide diversity for the whole species is amounted to 0.00227±0.00032 with the haplotype diversity *P* = 0.801 ± 0.040. The nucleotide diversity and haplotypic diversity for specimens from the mainland part of the Russian Far East are amounted to *P* = 0.503 ± 0.113, *π* = 0.00084 ± 0.00022. These values are close to the values of haplotype diversity for the COI gene described for *M. ikonnikovi* from South Korea (P = 0.5–0.8667) which are characterized by high genetic diversity of mitochondrial genes compared to other *Myotis* species (Park et al. 2019). The similar values of haplotype diversity have found for control region of two Northern American bat species *M. lucifugus* (P = 0.812–0.845) and *M. septentrionalis* (P = 0.827–0.910) (Johnson et al. 2015). At the same time the haplotype diversity of cyt b gene described for European *M. myotis* was amounted to *P* = 0.491 (Ruedi and Castella 2003), and for *M. dasycneme* was P = 0.335–0.868 (Andersen et al. 2018). On the other hand, the nucleotide diversity of *M. petax* is lower than that of *M. ikonnikovi* (π between 0.00163 to 0.00878) and is comparable with the nucleotide diversity of cyt b of *M. myotis* (π between 0.0003 to 0.0028) and *M. dasycneme* (π between 0.0004 to 0.0029) (Ruedi and Castella 2003, Andersen et al. 2018, Park et al. 2019).

A total of 9 COI haplotypes found in all specimens of *M. petax* studied including GenBank data (G1–9) but only 3 COI haplotypes detected in 18 *M. petax* individuals from the Russian Far East (G1–3). The G2 haplotype revealed at the first time.

**Figure 2.**
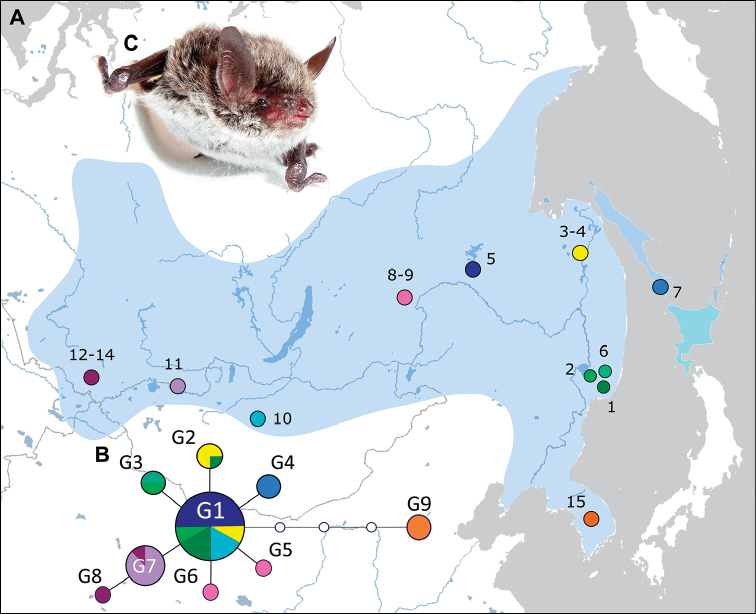
Distributional range and COI haplotypes of *Myotis petax***A** map showing approximate range and capture sites of *M. petax* (for this paper and previous studies) **B** median-joining network of COI haplotypes are colour-coded based on capture sites, circle size corresponds to number of samples **C***M. petax* (Russia, Buryatia Republic, 2014), photo by Denis V. Kazakov.

The relationship among a total of 9 haplotypes reflected in the median‐joining network (Fig. 2) revealed a close relationship between the all *M. petax* studied, expect the Korean bats which are more distantly related to other populations. The most common haplotype, G1, is observed in the waist territory from Baikal Lake to Pacific Ocean coast. It is found in 16 of the 44 specimens studied. Khabarovsky Krai and Primorsky Krai shared haplotype G2 which is found in the 4 individuals. The third haplotype observed in the Primorsky Krai is a G3 haplotype found in 2 specimens. Two individuals from Transbaikal Krai have two different haplotype G5 and G6, and 5 bats from the Sakhalin Island have G4 haplotype.

Haplotypes G7 and G8 form a separate branch on the network and are found only in 8 specimens from Tuva and the Altai. The G8 haplotype revealed in the one specimen from the Altai differed from G7 on one nucleotide substitution and from G1 on two nucleotide substitution. The spread of G7 and G8 is apparently coincides with the distribution of nominative subspecies.

The other differential branch on the network is a G9 haplotype differed from G1 on three nucleotide substitution. It is found only in 4 individuals from South Korea. The distinct subspecies for *M. petax* from Korea has not been described previously.

Most of the haplotypes represented in the samples are separated by G1 just one mutation creating a starlike network characteristic for expanding populations that have been through a bottleneck or been founded recently. However, COI gene is conservative and is not suitable for studying population events.

### Karyotype, differential staining and chromosomal polymorphism o*f M. petax* from the Far East

The conventional staining karyotypes of eight *M. petax* specimens from Primorsky Krai and Amur Oblast have no differences and shows 2n = 44 with the NFa = 52 (Table 1). There are composed of three large (1/2, 3/4, 5/6) and one small (16/17) metacentric pair, 17 acrocentric-subtelocentric autosomal pairs and one pair of sex chromosomes (X, Y). The X is a medium-sized biarmed chromosome. The small-sized Y chromosome is acrocentric and largely heterochromatic.

It was previously reported for the specimens from the Primorsky Krai the fundamental number of autosomal arms was 50 (Korablev et al. 1989). We already noted that variations of fundamental number in the different studies can be explained by different approaches to the taking into account short euchromatic arms on the seventh autosomal pair or the including the additional heterochromatic short arms on 24 or 25 pairs of acrocentrics in NFa (Kartavtseva et al. 2014, Gorobeyko and Kartavtseva 2019). While the karyotype of *M. petax* from South Korea (NFa = 52) showed short arms on 24 or 25 pair of acrocentric (Yoo and Yoon 1992), the all Far Eastern specimens studied have no short arms on these autosomal pairs. The image of *M. petax* chromosomes from the Primorsky Krai is not given and there is no mention of the presence or absence short arms on any autosomal pairs in the paper (Korablev et al. 1989).

The X chromosome is biarmed and it was not possible to determine whether this is a submetacentric or metacentric. At the same time the previously examined individuals from the Primorsky Krai have shown clearly a metacentric X chromosome. It is possible that these karyotypic differences are due to the methodological difficulties, such as the various spiralization of metacentric chromosomes or the lack of metaphase plates on the preparation often occurred in the analysis of chromosome suspensions obtained *in vivo*.

**Figure 3.**
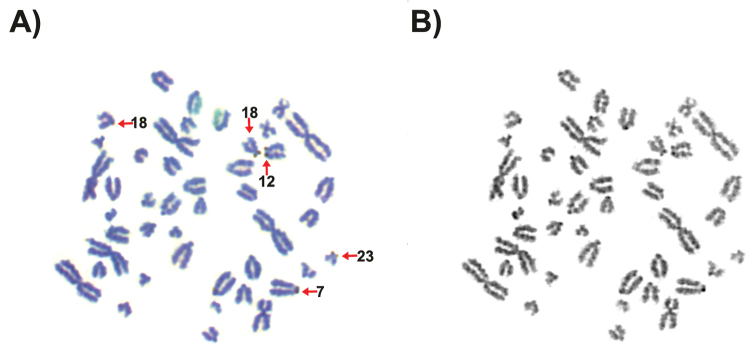
The sequential GTG- and AgNOR-banding of *Myotis petax* chromosomes **A** the AgNOR-banded karyotype of male 3259. Arrows indicate the NOR-bearing xcrocentric chromosome. Ordinal numbers indicate autosomal arm numbers revealed by GTG-banding **B** the GTG-banded karyotype of male 3259.

The patterns of NOR and the heterochromatic segments in karyotype of *M. petax* are described at the first time. Figure 3 demonstrated the sequential GTG- and AgNOR-banding of *Myotis petax* chromosomes. The distribution of active NORs in the four *M. petax* specimens is shown in Table 2. All four specimens showed active NORs in the minute short arms of chromosomes Nos. 7, 9, 10, 12, 13, 15, 18, 20, 21, 23-25.

**Table 2.** Distribution of nucleolus organizer regions: mean value of active NORs per chromosomal arm and cell. ID – identification number of specimen. No cells – number of cells analyzed. The numbers before ID (1, 2, 5) indicate sampling localities, the abbreviations see in Table 1.

ID	No cells	chromosomal arm no.																
		7	8	9	10	11	12	13	14	15	18	19	20	21	22	23	24	25
**1-3400**	**11**	0,41		0,5	0,27		0,14	0,09		0,27	0,86		0,55	0,27		0,27	0,27	0,09
**2-3259**	**20**	0,78		0,93	0,48		0,2	0,33		0,45	1		0,8	0,8		0,13	0,2	0,2
**5-3336**	**22**	0,16		0,38	0,13		0,19	0,17		0,27	0,36		0,33	0,38		0,38	0,14	0,2
**5-3338**	**63**	0,34		0,63	0,41		0,45	0,32		0,68	0,9		0,88	0,56		0,18	0,1	0,06

On average only 4.7 NOR per cell from 24 potential sites is detected that illustrated the low NOR activity of all the specimens studied. In many cells only one homologue of a chromosomal pair is shown to bear an active NOR. A similar low NOR-activity was shown for *M. myotis*, *M. capaccinii*, *M. bechsteinii* (Volleth 1987) and for *M. bombinus*, *M. longicaudatus*, *M. macrodactylus* (Ono and Obara 1994). All these species including *M. petax* have small multiple centromeric NORs on chromosomes.

*M. petax* is clearly differ on the number and localization of NORs as from the other Far Eastern *Myotis* species as from the sibling species *M. daubentonii*. The comparison of the NOR distributions in the karyotypes of the Far Eastern *Myotis* species is shown in Table 3. The NOR-distribution of the one Far Eastern species *M. sibirica* (*gracilis*) is still unknown. The conventional staining karyotype of this species (2n = 44, NFa = 50) was published by Kartavtseva and Dokuchaev (1998).

**Figure 4.**
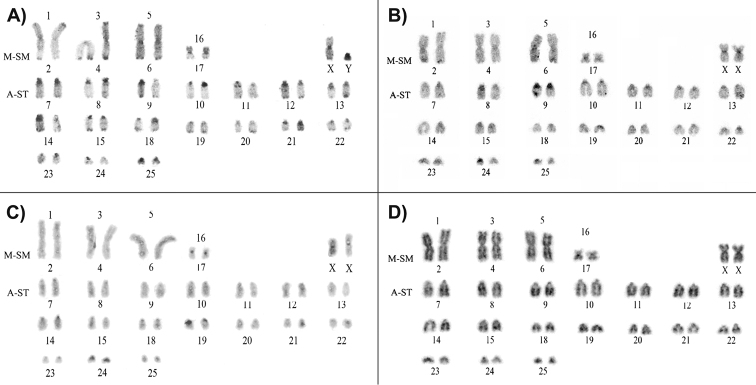
Comparison of C-banded karyotypes of far eastern *Myotis petax***A** CBG-banded karyotype of specimen 3259 (locality 2) **B** CBG- banded karyotype of 3865 (locality 1) **C** C-banded karyotype of specimen 3338 (locality 5) **D** GTG-banded karyotype of 3865 (locality 1). The abbreviations see in Table 1.

The amounts and localizations of heterochromatin bands on chromosomes of three *M. petax* from the Primorsky Krai and Amur region presented in Figure 4A–C and are clearly different.

**Table 3.** Distribution of NORs in karyotypes of the Far Eastern *Myotis* species. * – distribution of NORs of European species, *Myotis daubentonii*, is shown to comparison with distribution of NORs in karyotype of *M. petax*. The abbreviations see in Table 1. **cmc** – centromere-cap NORs, ST – subtelocentric chromosome.

Species	2n	NFa	*X*	*Y*	Chromosome arm no.																	NOR	Source
					7	8	9	10	11	12	13	14	15	18	19	20	21	22	23	24	25		
*Myotis bombinus*	44	52	M	A	+	+	+	+	+	+	+	+	+		+			+				11 cmc	Ono and Obara 1994
*M. longicaudatus*	44	52	M	ST		+	+	+	+		+	+	+	+	+	+	+	+	+			13 cmc	Ono and Obara 1994
*M. ikonnikovi*	44	52	M	A	+						+	+						+	+			5 cmc	Ono and Obara 1994
*M. macrodactylus*	44	52	M	A										+	+	+	+	+	+			6 cmc	Ono and Obara 1994
*M. petax*	44	52	M-SM	A	+		+	+		+	+		+	+		+	+		+	+	+	12 cmc	this study
M. daubentonii*	44	52	SM	SM		+	+	+														3 cmc	Volleth 1987, Volleth and Heller 2012

1) The male *M. petax* (3259) from Spasskaya Cave (locality 2) showed centromeric heterochromatic bands on most of chromosomal pairs. The one or two homologues in chromosome pairs Nos. 7–10, 12–14 and 25 bore large centromeric heterochromatic segment. Small but distinct telomeric heterochromatic bands are found on all biarmed chromosomal pairs and seven acrocentric pairs from 7 to 22. Large intercalary heterochromatic segments are located on chromosome 8, 11 and 18. A heteromorphism in localization of heterochromatin blocks found in nine autosomal pairs 8–12, 14, 18, 21, 24.

2) The female *M. petax* (3865) from the Primorsky Velican Cave (locality 1) showed centromeric heterochromatic bands on most of the acrocentric pairs, on the metacentric pair 16/17 and X chromosome. The large heterochromatic centromeric segments are found in 8 and 9 autosomal pairs. The telomeric heterochromatic segments are presented on all biarmed chromosomal pairs and acrocentric pairs Nos. 11 and 21. A heteromorphism in localization of heterochromatin blocks is found in four autosomal pairs 8, 25 and 16/17. There were no intercalary heterochromatic bands in karyotype of *M. petax* from the Primorsky Velican Cave. The GTG-banded karyotype of 3865 showed in Figure 4D.

3) In karyotype of the female *M. petax* (3338) from Zeya (locality 5) small and slightly stained heterochromatic centromeric bands are found on nine acrocentric pairs from 7 to 25, metacentric pair 16/17 and X chromosome. Three autosomal pairs 7, 14 and 22 showed a heteromorphism on amount heterochromatic material. This specimen had no telomeric or intercalary heterochromatic bands.

The distinct telomeric heterochromatic segments found on several chromosomes of both individuals from the Primorsky Krai were previously described only for the Chinese *Myotis* species such as *M. altarium* Thomas, 1911 (Li et al. 2007), M. cf. siligorensis (published as “*M. dividii*), M. cf. daubentonii (Peng et al. 2011), *M. fimbriatus* (Peters, 1871) (Wang et al. 2009). The intercalary heterochromatic segments were observed in karyotypes of Eurasian *Myotis* species (Volleth and Heller 2012), but no one have intercalary heterochromatin bands on acrocentric pairs Nos. 8, 11, 18 found in the specimen 3259.

All individuals studied had the heteromorphic chromosome pairs. The similar intraspecific heteromorphism of several heterochromatic segments was previously observed in a few Eurasian *Myotis* species (Harada and Yosida 1978; Volleth and Heller 2012). Intraspecific polymorphism of the several heterochromatic segments in karyotypes of a few Eurasian *Myotis* species is illustrated in the Table 4. Nevertheless, a variability of the heterochromatic material as found in karyotype *M. petax* is not typical for the most of the Eurasian *Myotis* species. We have already noted the same significant polymorphism in the amount and location of the heterochromatin bands in the karyotype of two *Pipistrellus*-like species: *Pipistrellus abramus* (Temminck, 1840) and *Vespertilio sinensis* Peters, 1880 (Ando et al. 1980; Harada et al. 1987; Ando et al. 1987; Ono and Obara 1994; Ono and Yoshida 1997; Lin et al. 2002; Wu et al. 2009; Gorobeyko and Kartavtseva 2019).

The individuals differing in the amounts and localizations of heterochromatin bands on chromosomes are also belonged in different COI haplotypes. The specimen 3331 from Amur Oblast is showed G1 haplotype, while the bats 3259 and 3865 from the Primorsky Krai are belonged to G3 and G2, respectively. Nevertheless, the number of *M. petax* individuals studied and the differences between the COI haplotypes are insufficient to draw conclusions regarding the relationship between chromosomal and COI variability.

**Table 4.** Intraspecific variations of heterochromatic material in karyotypes of *Myotis* species. specimen – identification number (ID) of specimen. The numbers before ID (1, 4, 5) indicate sampling localities, the abbreviations see in Table 1. Symbols: o – totally euchromatic chromosomes, • – totally heterochromatic chromosomes. + – small heterochromatic band in vicinity of the centromere on both homologues in pair, ++ – large heterochromatic band in vicinity of the centromere on both homologues in pair, x/xx – heteromorphic heterochromatic bands in vicinity of the centromere: small and absent / large and small or absent. tel – heterochromatic segment in vicinity of the telomere, int – interstitial heterochromatic band, arm – heterochromatic secondary arm. ***Bold Italic*** – heteromorphic chromosomal pair. The abbreviations see in Table 1.

**Specimen/Species**	**2n**	**NFa**	**Chromosome arm no.**																					**X**	**Y**	**Sourse**
			1/2	3/4	5/6	16/17	7	8	9	10	11	12	13	14	15	18	19	20	21	22	23	24	25			
**1-3338**	44	50	o	o	o	+	x	o	o	+	o	+	o	x	o	+	+	o	o	x	+	++	o	+	-	**Present study**
**4-3259**	44	50	tel	tel	+, tel	+, tel	++, tel	+, ***int***	xx, tel	xx	+, ***int***	xx, tel	+, tel	xx	+, tel	+, ***int***	+, tel	o	xx	+, tel	+	x	++	+	•, A	**Present study**
**5-3865**	44	50	tel	tel	tel	+, ***tel***	o	xx	++	+	+, tel	+	+	o	+	+	+, tel	+	+, tel	+	+	+	x	+	-	**Present study**
***M. m. bulgaricus***	44	52	+	+	+	+, int	+	+	+	+	+	+	+	+	+	+	+	+	+	+	+	+	+, ***arm***	+	•, SM	Volleth and Heller 2012
***M. daubentonii***	44	52	+	+	+	+	+	+	+	+	+	+	+	+	+	+	+	+	+	+	+	+	+	int , ***int***	•, ***SM/ST***	Volleth and Heller 2012
***M. ikonnikovi***	44	52	++	++	++	++	++	+	++	++	++	++	++	+	+	+	+	++	+	+	x	x	arm, ***A/M***	++	-	Harada and Yoshida 1978
***M. macrodactylus***	44	52	xx	+	xx	+	+	++	++	+	++	+	+	+	++	++	+	+	+	++	+	+	arm, ***SM/M***	++	•, A	Harada and Yoshida 1978

## Conclusion

The COI barcoding showed the presence of only 3 COI haplotypes (G1–3) in the Russian Far East from 9 COI haplotypes (G1–9) found in *M. petax*. The G2 haplotype detected at the first time. This species showed to have the low nucleotide variability with the prevalence of the central, the most abundant haplotype. The distances between individuals do not exceed 0.8%.

The chromosomal characteristics of *M. petax* from the Russian Far East are clarified. The distributional patterns of NOR and heterochromatic segments on the chromosomes *M. petax* are described at the first time. The number and localization of NOR in karyotypes of sibling species *M. petax* and *M. daubentonii* is different and can be used as diagnostic feature. The significant intraspecific variability in the heterochromatin distribution of revealed in Far Eastern *M. petax* was not described for the genus *Myotis*, but it had been found in other vespertilionid species.

## Supplementary materials

Supplementary material 1 The other collecting data for *Myotis petax* Data type species data https://binary.pensoft.net/file/463921This dataset is made available under the Open Database License (http://opendatacommons.org/licenses/odbl/1.0/). The Open Database License (ODbL) is a license agreement intended to allow users to freely share, modify, and use this Dataset while maintaining this same freedom for others, provided that the original source and author(s) are credited.Uliana V. Gorobeyko, Irina V. Kartavtseva, Irina N. Sheremetyeva, Denis V. Kazakov, Valentin Yu. Guskov
